# Interventions for mitigating occupational stress for professional dementia caregivers in residential aged care: A systematic review with meta-analysis

**DOI:** 10.1177/14713012231220963

**Published:** 2023-12-08

**Authors:** Hayley Antipas, Jeanette Tamplin, Tanara Vieira Sousa, Felicity A. Baker

**Affiliations:** Creative Arts and Music Therapy Research Unit, Faculty of Fine Arts and Music, 2281 The University of Melbourne, Australia; Creative Arts and Music Therapy Research Unit, Faculty of Fine Arts and Music, 2281 The University of Melbourne, Australia; Royal Talbot Rehabilitation Centre, Australia; Creative Arts and Music Therapy Research Unit, Faculty of Fine Arts and Music, 2281 The University of Melbourne, Australia; Creative Arts and Music Therapy Research Unit, Faculty of Fine Arts and Music, The University of Melbourne, Australia. Norwegian Academy of Music, Norway

**Keywords:** caregivers, dementia, meta-analysis, occupational stress, workforce

## Abstract

**Objective:**

Occupational stress in professional dementia caregivers in residential aged care facilities has adverse effects on care quality, caregivers’ health, and workforce sustainability. The purpose of this study was to examine the evidence regarding interventions to mitigate occupational stress for this population.

**Methods:**

A systematic review of CINAHL, PsycINFO, PubMed and MEDLINE databases was conducted to identify original RCT research reporting on stress interventions, published in English between 1995 and March 2022. Search results were screened by two independent reviewers. Quality and risk of bias were appraised using the Downs and Black Checklist and Risk of Bias by two reviewers. Meta-analysis and subgroup analysis examined the pooled intervention effects on stress compared to control.

**Results:**

10 studies met the inclusion criteria, and these reported on 15 interventions and 28 outcomes from 92 facilities, involving 1,397 caregivers. We found a small and insignificant effect of interventions on caregiver stress (g = −.27, *p* = .16). Heterogeneity was partially explained by subgroup analysis. Interventions can mitigate stress and burden not attributed to client behaviour (*n* = 3) (g = −.85, *p* < .001), and improve caregivers’ self-efficacy (*n* = 4) (g = −.35, *p* = .07). We were unable to determine the most effective type of intervention, although organisation focused interventions showed the greatest potential (g = −.58, *p* = .08).

**Conclusion:**

Interventions that improve caregivers’ personal and organisational resources can reduce non-client associated stress and burden and increase self-efficacy. Aged care providers are recommended to prioritise education with organisational support interventions. Research on longitudinal effects and high-risk caregivers is required. Limitations are discussed.

**Prospero Registration Number:**

CRD42022313715 (registered April 2022).

## Impact statement

Residential aged care employers can improve caregivers’ self-efficacy and reduce stress and burden with interventions that enhance personal and organisational resources. Generalised interventions may not be effective to reduce burnout, thus change mechanisms and individualised interventions require further research. Reducing occupational stress is crucial for sustainability of the caregiver workforce.

## Introduction

Worldwide more than 55 million people are living with dementia, a number expected to rise exponentially over the coming decades (World Health Organisation, 2021). Dementia is a collective term for a range of neurodegenerative conditions that can affect all aspects of a person’s physical and psychological health and day to day living. People with a diagnosis of dementia represent more than half of those residing in permanent care and nursing homes, herein collectively referred to as residential aged care facilities (RACFs) (Dementia Australia, 2023; Freedman et al., 2021). In 2021 care for people living with dementia in RACFs cost Australia alone $4 billion (Brown et al., 2022). Thus, this is of significant health and economic concern. Professional caregivers play a vital role in providing clinical, social, emotional, and relational support for people living with dementia in RACFs. Despite being a fulfilling role, the challenges of supporting people living with dementia can lead to high levels of stress and poor job satisfaction (Costello, Walsh, et al., 2019) which compromises both caregiver and care recipient health and wellbeing (Le Fevre et al., 2003; Rajamohan et al., 2019).

### Occupational stress in professional dementia caregivers

While stress is a term most people are familiar with colloquially it is rarely defined in the literature and often used inconsistently and interchangeably with terms including distress, strain, burden, burnout, and mental wellbeing (Kemeny, 2003). While stress and burnout differ in that burnout is typically associated with chronic stress, they are often conceptualised in the same framework (Maslach & Jackson, 1981). The current study therefore adopts an integrative view, considering stress a negative subjective experience which may be described as feeling “stressed out” (McEwen, 2005). This experience is typically associated with the persistent or long-term stress which creates a cumulative dysregulation and toll on the brain and body (Beckie, 2012), and can lead to burnout (Maslach et al., 2001). Stress is associated with reduced regulatory capacity and negative physical health, mental health and behavioural outcomes for caregivers (Koolhaas et al., 2011; Le Fevre et al., 2003), employee burnout (Woodhead et al., 2016), job dis-satisfaction (Costello, Walsh, et al., 2019), reduced quality of care (Zimmerman et al., 2005), employee turnover (Rajamohan et al., 2019), and significant economic costs (World Health Organisation, 2021).

**Figure 1.**
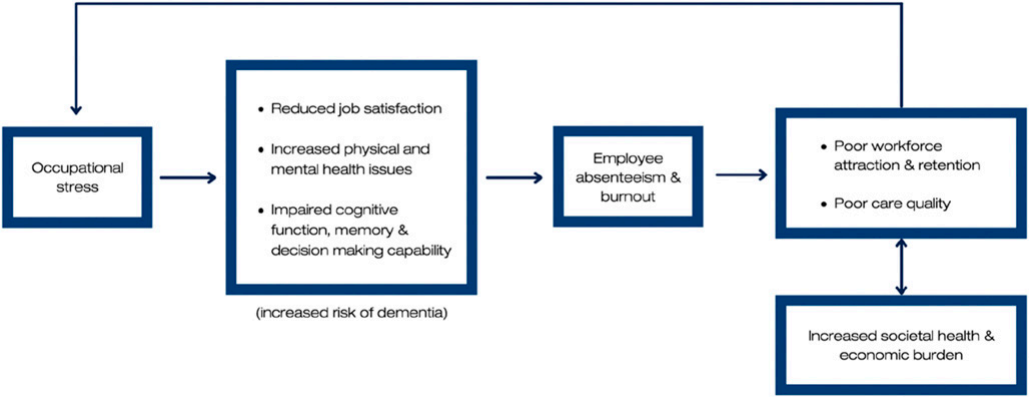
Impact of occupational stress in professional dementia caregivers in residential aged care facilities. Note. Researcher’s visual construction of the impact of occupational stress on professional caregivers, people living with dementia and society through: job satisfaction (Costello, Walsh, et al., 2019) caregivers’ health (Koolhaas et al., 2011; Le Fevre et al., 2003), risk of dementia (Wang et al., 2012), cognitive function (McEwen & Sapolsky, 1995), absenteeism and retention (Rajamohan et al., 2019), burnout (Woodhead et al., 2016), care quality (Zimmerman et al., 2005), and societal burden (World Health Organisation, 2021).

Professional caregivers are paid employees working at RACFs (not client family members) who may have a certificate III or IV work-related qualification (Kostas Mavromaras et al., 2017) and are not clinical professionals such as Enrolled or Registered Nurse. Caregivers are primarily responsible for supporting people with dementia with their everyday living including personal care. The RACF environment and job demands of caregiving have been described as *“fertile ground for persistent stress”* (Pearlin et al., 1990, p. 1) Stress in this population is complex and multifaceted as organisational, client and personal factors can both contribute to and moderate stress (Chamberlain et al., 2017; Cohen-Mansfield, 1995). The detrimental impact of occupational stress escalates in a vicious cycle where outcomes of stress contribute to increasing stress (Figure 1). Interventions to mitigate the effects of occupational stress are thus crucial to the provision of high-quality care for people living with dementia in RACFs whilst simultaneously addressing the industry’s workforce retention challenges and societal burden.

### Occupational stress interventions

  Multiple factors have been associated with professional caregiver stress and burnout. Improved organisational culture factors including leadership, feedback, resources, care environment, and social support are frequently associated with reduced burnout (Chamberlain et al., 2017; Costello, Walsh, et al., 2019; Woodhead et al., 2016). Staffing levels (Costello, Walsh, et al., 2019), lower physical and mental health (de Rooij et al., 2012), personal characteristics including primary language spoken (Chamberlain et al., 2017; Costello, Walsh, et al., 2019), caregivers’ socioeconomic status (Chandola & Marmot, 2010), self-efficacy (Duffy et al., 2009), and perceived mastery and control at work (Testad et al., 2010) are associated with burnout. Notwithstanding, job demands associated with client’s behaviour that caregivers may find challenging, sometimes referred to as Behavioural and Psychological Symptoms of Dementia (BPSD), are related to burnout (Woodhead et al., 2016).

This suggests interventions that focus on reducing client related stressors, improving caregivers’ capacity to manage and cope with client and organisational stressors, and improving personal and organisational stress moderators may be effective in mitigating stress and burnout for caregivers. This theory aligns with current evidence that suggests stress interventions should focus on optimising the moderating factors which promote adaptation and mitigate the negative effects of persistent or chronic stress (McEwen, 2005). Within a broader professional population, interventions designed to increase employees’ personal resources (self-efficacy, coping, wellbeing) or job skills (knowledge and capacity) have been found to produce significant effects on stress reduction (Richardson & Rothstein, 2008). However, it is unclear what interventions may be most effective to reduce stress in professional dementia caregivers in RACFs. Further investigation is required to understand the types of interventions and their impact on mitigating professional caregiver stress and related psychological experiences.

### This study

The effect of interventions to mitigate professional caregivers’ occupational stress when supporting people living with dementia in RACFs has not been systematically investigated. Mitigating stress in this population may be key to future-proofing the industry and maintaining the mental health of the workforce, which in turn ensures high-quality care for people living with dementia and reduces societal burden. Given intervention type plays a moderating role in stress mitigation (Richardson & Rothstein, 2008), and stress in professional dementia caregivers is complex (Chamberlain et al., 2017; Cohen-Mansfield, 1995), this study seeks to summarise the existing evidence and inform future research by examining: what interventions are used to mitigate professional caregivers' occupational stress experience in RACFs and how effective they are. The following null hypotheses were posed: 1) intervention does not mitigate professional caregivers’ occupational stress compared to usual practice, and 2) there is no difference between the type of intervention and its effect on mitigating professional caregivers’ occupational stress.

## Method

### Search and selection of studies

CINAHL, PsycINFO, PubMed and MEDLINE databases were systematically searched to identify peer reviewed articles published in English between 1995 and March 2022 that trialled an intervention to mitigate occupational stress in professional caregivers who support people living with dementia in RACFs. Given the heterogeneity and inconsistent use of terminology, for the purpose of this review, studies that refer to professional caregiver stress, burden, burnout, wellbeing, anxiety, depression, and/or coping were considered to address the focus topic of occupational stress. The Boolean search phrase used was: (stress or burden or burnout or strain or distress or anxiety or depression or eustress or coping or wellbeing or well-being or “mental health”) AND (dementia or Alzheimer*) AND (carer or caregiv* or “personal care assistant” or “personal care attend*” or “aged care staff” or “care staff” or “aged care support worker” or “care worker” or “personal care aide” or “care assistant” or “personal support aide” or “care aide”) AND (professional* or formal or employ* or staff or paid or worker) AND (“residential aged care” or “nursing home” or “elder care” or “long-term care” or “care home”).

Search results were uploaded and screened using Rayyan software (Ouzzani et al., 2016), where duplicates were removed, and the titles and abstracts of unique studies were screened against the eligibility criteria by two independent reviewers. After screening titles and abstracts, full texts of any articles identified as potentially eligible were retrieved and screened to determine eligibility. Conflicts which could not be resolved through discussion between the two reviewers were referred to a third reviewer. The search was supplemented with a hand search of the reference lists of eligible studies.

### Eligibility

Professional caregivers were considered paid employees working at RACFs (not client family members) who do not appear to have a nursing or allied health qualification and are primarily responsible for supporting people with dementia with their activities of daily living including personal care. Studies were included where >50% of the participants met these criteria. We excluded studies that: were review studies; were not randomised controlled trial design; did not provide a sufficient description of the intervention to allow replication; or measured non-work-related anxiety and/or depression.

### Data extraction

A researcher designed form was used to extract data from each of the eligible studies for assessment of quality, risk of bias and to conduct the data analysis. Only outcome data measuring caregiver stress was extracted. Outcome data relating to resident outcomes, such as symptoms of dementia, were not collected. Data was extracted by the first author and spot-checked for accuracy by a second author.

### Quality appraisal and risk of bias

Eligible studies were evaluated for quality and risk of bias independently by two authors with discrepancies discussed referring to the full text until a consensus was reached. Where a consensus could not be reached, a third author was consulted.

#### Downs and Black quality appraisal

The quality of studies was appraised using the Downs and Black checklist (Downs & Black, 1998). This 27-question checklist assesses the quality of reporting, external validity, internal validity bias and confounding, and statistical power. It has been shown to have high internal consistency (α = .89) and good validity (α = .54). The statistical power question carries a possible five points and has been modified to a single point score where the study has sufficient power to detect a clinically important effect, resulting in a maximum total score of 28, which is an approach frequently used (O’Connor et al., 2015). Each study was assigned a quality grade of ‘excellent’ (24-28 points), ‘good’ (19-23 points), ‘fair’ (14-18 points), or ‘poor’ (<14 points).

#### Risk of bias 2.0 assessment

Assessment for risk of bias was performed using the Cochrane recommended Risk of Bias version 2.0 (RoB2) for individually or cluster randomised trials as relevant (Sterne et al., 2019). RoB2 includes up to 28 signalling questions to assess the risk of bias in the trial design, conduct, and reporting on the effect of assignment to intervention. Based on the responses an algorithmically generated rating of ‘low’ or ‘high’ risk of bias, or ‘some concerns’ (moderate risk) was allocated to each study.

### Data analysis

Data was pooled with excel and analysed with RevMan 5.4 (Review Manager, 2020). Hedges-g standardized mean difference (SMD) with 95% confidence intervals (CI) was calculated with change scores, which is considered trustworthy even when sample sizes are small (Borenstein et al., 2021). Meta-analysis using change scores removes between-person variability and may be more efficient and powerful than comparison of post-intervention scores (Deeks et al., 2022). Where reported, adjusted change scores that account for baseline measurements as a covariate were used and synthesised using a random effects model meta-analysis. Otherwise change scores were requested from the authors via email. No authors provided the requested data, thus change scores and missing standard deviations (SD) were calculated in accordance with the cochrane recommendations (Higgins et al., 2022). Change scores were calculated by subtracting the pre mean from the post mean. One study (Fukuda et al., 2018) was reported in sufficient detail for correlation coefficients to be calculated, with the average correlation coefficient calculated and used to impute SD for the remaining studies and outcomes (r = .723).

For studies with multiple intervention arms, results were pooled using RevMan calculator to produce a combined intervention effect, which avoids multiplicity in the analysis. Where multiple time points were reported, the outcomes closest to the end of the intervention period were selected to be comparable with other studies which only reported one post-intervention outcome. To deal with multiplicity from multiple outcome measures, average effect size was used. Finally, a negative change score was considered a beneficial intervention effect on stress (mitigation). Outcomes where a positive change score represents a beneficial effect on stress (e.g. an increase in personal accomplishment), were multiplied by −1 to maintain consistency across the data set.

Statistical variation across studies was measured using I^2^ and subgroup analysis was used to further investigate heterogeneity and test hypothesis two. Given the small sample size, random effects with pooled estimates of τ^2^ were calculated for subgroup analysis with Chi^2^ analysis of variance (Q) intended to compare the mean effect of interventions between subgroups. Subgroup analysis was based on a) outcome domain and b) intervention type.

Sensitivity analysis was used to test the robustness of decisions made throughout the design and analysis process to determine if they changed the results. Kappa coefficients for inter-rater agreement and descriptive statistics were calculated using SPSS 28 software (Corp, 2021) and Excel 16 respectively. A significance level of *p* < .05 was used.

## Results

### Search and study selection

**Figure 2.**
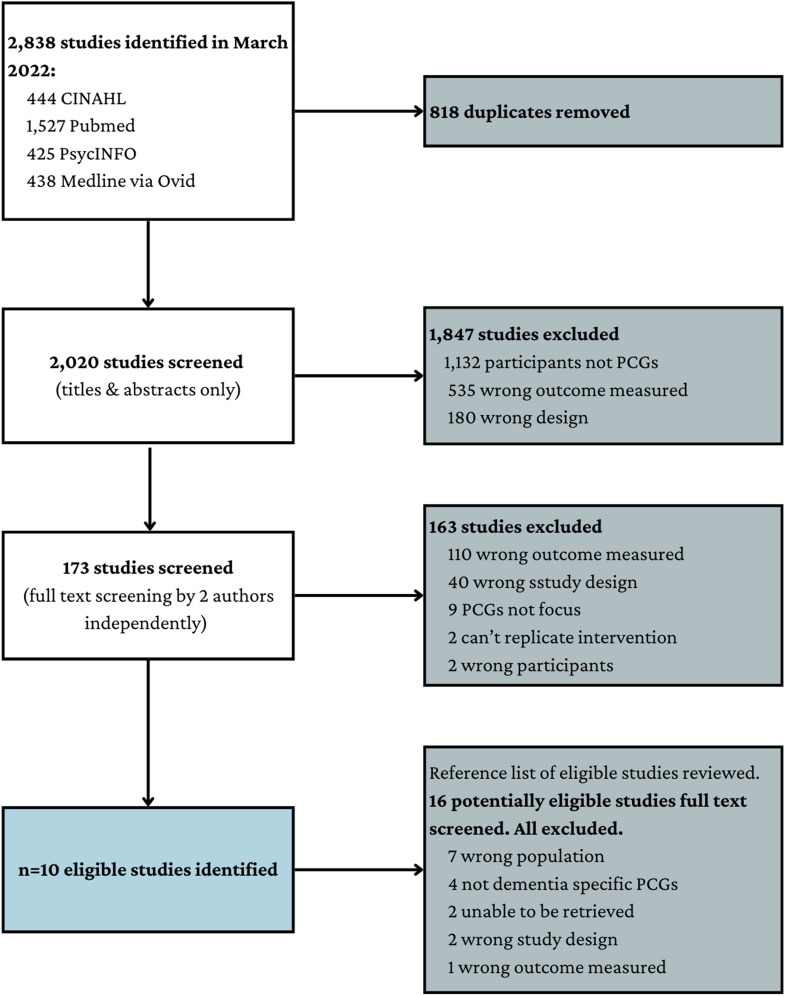
PRISMA flow diagram of study screening and selection.

Database searches were conducted on 29^th^ March 2022. A total of 2,838 references were retrieved and 818 duplicates were removed, resulting in a library of 2,020 unique studies. After screening against the eligibility criteria independently by two reviewers, 10 studies met all inclusion criteria and were included in the review (Figure 2). Studies were primarily excluded because they focused on family caregivers or persons with dementia outcomes only or utilised an ineligible study design. Inter-rater agreement was high (κ = .936, SE = .026, 95% CI = .885 – .987). Manual citation checking revealed 16 additional studies that possibly met the eligibility criteria; however, all were excluded upon full text review. In total, 10 studies were eligible for inclusion in the review.

### Study characteristics

The 10 included studies reported on 15 interventions and 28 outcomes. Across the studies, interventions were delivered in 92 RACFs, and included 1,397 professional caregivers. Within individual studies, reported samples of professional caregivers ranged from 19 to 368 (M = 174.6, SD = 138.04). Six studies provided demographics on professional caregivers, indicating on average they were 43.3 years old (SD = 3.93) and mostly female (*n* = 999, 88.15%). Studies were implemented in seven countries with cluster randomisation (*n* = 6) most common (Table 1

**Table 1.** Characteristics of studies included in review.

Study	Design	Country	Participants			PCG characteristics	Duration (months)	Intervention	Outcome(s)				Quality	Risk of bias
			PCG	RACF	PWD				Control	Domain	Measure	Rater		
Davison et al. (2007)	Cluster randomised, 3-parallel arms	Australia	*n* = 90	*n* = 6	*n* = 113	Age m = 45 (SD = 11) years. 90% female	Not reported	a. Caregiver focused: Job-skills training b. Organisational: Peer support program	Care as usual	i. Burnout (EE, DP, PA subscales) ii. Self-efficacy	i. Maslach burnout inventory ii. Self-efficacy of dementia care	Self-reported, not masked	Poor	High risk
Frank et al. (2004)	Individually randomised, placebo controlled trial	Australia and New Zealand	Not reported	*n* = 14	*n* = 279	Not reported	3	Client focused: Risperidone drug treatment	Placebo	Stress	Modified strain in nursing care assessment scale	Self-reported, not masked	Fair	Some concerns
Fukuda et al. (2018)	Cluster quasi-randomised, 2-parallel arms	Japan	*n* = 357	*n* = 18	n/a	Age m = 37.2 (SD = 12.8) years. 77% female	1	Caregiver focused: Job-skills with printed educational material	Care as usual	Burnout (EE, DP, PA subscales)	Maslach burnout inventory (Japanese)	Self-reported, not masked	Good	Some concerns
Halek et al. (2020) ^a^	Cluster randomised, stepped wedge	Germany	*n* = 233	*n* = 12	*n* = 465	Not reported	7	Organisational: Case conferencing	No control	i. Burnout (total burnout, client-related, personal and work-related) ii. Stress	i. Copenhagen Burnout inventory ii. Dementia specific burden instrument	Self-reported, not masked	Excellent	Some concerns
Kuske et al. (2009)	Cluster randomised, 3-parallel arms	Germany	*n* = 96	*n* = 6	*n* = 210	Age m = 44 (SD = 7) years. 94% female	3	a. Caregiver focused: Job-skills training b. Caregiver focused: personal relaxation	Care as usual	Burnout (EE, DP, PA subscales)	Maslach burnout inventory	Self-reported, not masked	Good	Some concerns
McCabe et al. (2015)	Cluster randomised, 4-parallel arms	Australia and New Zealand	*n* = 204	*n* = 16	*n* = 187	Age m = 43 (SD = 13.4) years. 87% female	3	a. Caregiver focused: Job-skills training b. Organisational: Clinical support program+training with clinical support	Care as usual	i. Stress ii. Self-efficacy	i. Carer stress scale ii. Self-efficacy of dementia care	Self-reported, not masked	Good	Some concerns
Moyle et al. (2013)	Individually randomised, 2-parallel arms	Australia	*n* = 19	*n* = 1	n/a	Age m = 49 (range 23–63) years. 100% female	1	Caregiver focused: personal massage	Active control, silent resting	i. Stress (D-bp and S-BP) ii. Anxiety	i. Systolic and diastolic blood pressure ii. Faces anxiety scale	Independently, not masked	Fair	Some concerns
Santagata et al. (2021)	Individually randomised, 2-parallel arms	Italy	Not reported	*n* = 2	*n* = 52	Not reported	3	Client focused: doll therapy	Care as usual	Burden	Gruetzner scale	Self-reported, not masked	Good	Some concerns
Sautter et al. (2021) a	Individually randomised, 2-parallel arms	USA	*n* = 30	Not reported	*n* = 10	Not reported	3	Client focused: Personalised computer engagement	Care as usual	Stress	Perceived stress scale	Self-reported, not masked	Poor	High risk
Zwijsen et al. (2015) ^a^	Cluster randomised, stepped wedge	The Netherlands	*n* = 368	*n* = 17	n/a	Age m = 42 (SD = 12) years. 97% female	24	Caregiver focused: Job-skills education using GRIP program	Care as usual	i. Burnout (EE, DP, PA subscales) ii. stress iii. Wellbeing	Maslach burnout inventory (Dutch)	Self-reported, not masked	Fair	High risk

*Note.* EE = emotional exhaustion. DP = de-personalisation. PA = personal accomplishment.

^a^Insufficient data to include in effect size or subgroup meta-analysis.

). The mean study length was 5.33 months (SD = 7.21), although this was reduced to three months (SD = 1.85) when an outlier 24-month study (Zwijsen et al., 2015) was removed.

#### Quality and risk of bias

Using the modified Downs and Black checklist (Downs & Black, 1998), studies were rated as good (*n* = 4), fair (*n* = 3), poor (*n* = 2) and excellent (*n* = 1) quality. Risk of bias assessment identified that most studies had *some concerns* about risk of bias (*n* = 7) (see Table 1). There was poor initial agreement between reviewers for the quality assessment (κ = .043, *p* = .562) and risk of bias assessment (κ = .094, *p* = .679).

#### Outcome domain

Most studies (*n* = 9) contained multiple outcome measures*,* which when pooled resulted in a total of 28 outcomes (Table 1). All studies examined psychological stress using self-reported measures, while one study additionally employed a physiological measure (Moyle et al., 2013). The Maslach Burnout Inventory (Maslach et al., 1997) was most used (4 studies). Studies generally reported good internal consistency of outcome measures used (α = .64–.95), though consistency was not available for six outcomes. Data from 11 outcomes (3 studies), were insufficient to be included in the meta-analysis (Halek et al., 2020; Sautter et al., 2021; Zwijsen et al., 2015). In total, 17 outcomes (7 studies) were included in the meta-analysis.

#### Type of intervention

All studies compared at least one intervention group against a control group, with a total of 15 interventions across the 10 eligible studies. Most utilised a ‘care as usual’ (or usual practice) control (*n* = 8) (Table 1). Intervention types were categorised based on the overarching change focus (Table 2). Despite the distinct categories, all organisation focused interventions also included caregiver education. Insufficient data was reported for three interventions (*n* = 3), to be included in the meta-analysis (Halek et al., 2020; Sautter et al., 2021; Zwijsen et al., 2015). In total, 12 interventions (from 7 studies) were included in the analysis.

**Table 2.** Intervention categories of included studies.

Intervention type (Category)	Number of interventions	Number of studies (n)
Organisation focused	5	3
Caregiver education focused	5	5
Client focused	3	3
Caregiver therapeutically focused	2	2

### Effect size

**Figure 3.**
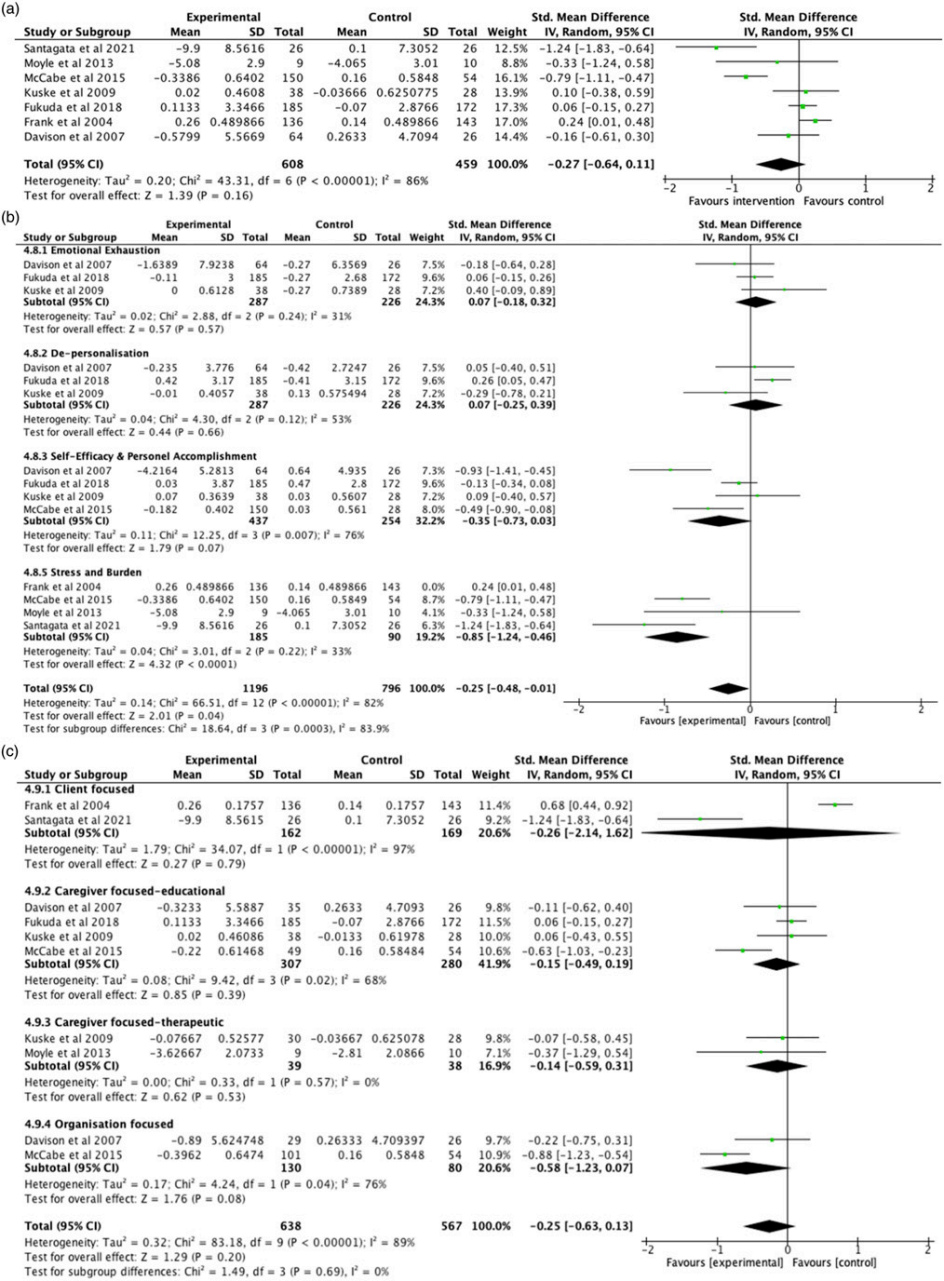
Results of meta-analysis across all eligible studies on the effect of intervention compared to control on professional caregiver stress. (a) Effect size for main meta-analysis. (b) Subgroup analysis by outcome domain. (c) Subgroup analysis by intervention type.

A small and insignificant pooled intervention effect on caregiver stress and burnout compared to control was observed (*g* = −.27, CI = −.64, .11, *p* = .16) (Figure 3(a)). There was considerable heterogeneity based on analysis of between study variance (I^2^ = 86%) which could not be accounted for by sampling error (*Q* = 43.31, df = 6, *p* < .001). Sensitivity analysis examined the impact of each individual study on heterogeneity. One study accounted for 9% of the variability (McCabe et al., 2015), although the direction and magnitude of the effect on stress was relatively stable (*g* = −.14, 95% CI = −.48, .19, I^2^ = 77%, *p* = .40). Thus, all studies with complete data were included in the effect size meta-analysis (*n* = 7). Overall, a small and insignificant intervention effect compared to usual practice on caregiver occupational stress was not sufficient to reject the primary null hypothesis. Given high heterogeneity indicates the presence of moderators, subgroup analysis to further test the primary hypothesis was justified.

### Outcome domain moderators

A large and significant intervention effect was observed on the subdomain of stress and burden (g = −.85, 95% CI -1.24, −.46, I^2^ = 33%, *p* < .001). A small intervention effect approaching significance was observed on self-efficacy and personal accomplishment, albeit with considerable heterogeneity (g = −.35, 95% CI -.73, .03, I^2^ = 76%, *p* = .07). No other significant intervention effects were observed (Figure 3(b)). Sensitivity analysis determined that an outcome measure that specifically measured stress related to coping with client behaviour introduced a considerable amount of heterogeneity (I^2^ = 92%) thus this outcome and study were excluded from the synthesis (Frank et al., 2004). The outcome domain measured accounted for much of the observed heterogeneity in the primary effect size analysis, yet confounders were not able to be determined due to the small number of studies in each subgroup, thus results are cautiously interpreted (Oxman & Guyatt, 1992). It was not possible to compare variance between subgroups as subgroups did not contain independent data (Deeks et al., 2022).

Given these results we can reject the primary null hypothesis that intervention does not mitigate professional caregivers’ occupational stress compared to usual practice. It appears that, in comparison to usual care, interventions can mitigate stress and burden that is not specifically attributed to coping with client behaviour and can possibly improve self-efficacy.

### Intervention type moderators

A medium intervention effect was found for organisation-focused interventions on caregiver stress, and this was approaching significance (g = −.58, 95% CI -1.23, .07, I^2^ = 76%, *p* = .08). This result should be considered cautiously given only three interventions (*n* = 2) were able to be synthesised and heterogeneity was not able to be explained by sensitivity analysis. No significant effects were observed for other intervention types (Figure 3(c)). While intervention type appeared to account for a small amount of the observed heterogeneity, confounders were not able to be determined due to the small number of studies available in each subgroup and we were unable to determine the size of the variance between subgroups due to subgroups not containing independent data (Deeks et al., 2022).

With no significant results for this subgroup analysis, we are unable to reject the null hypothesis that there is no difference between the type of intervention and its effect on mitigating professional caregivers’ occupational stress. While it is possible that organisation-focused interventions, in comparison to usual care, may mitigate professional caregiver stress, this was not statistically confirmed.

## Discussion

Professional caregivers who support people living with dementia in RACFs are exposed to high levels of occupational stress which compromises both caregiver and care recipient health and strains the aged care industry and economy (Brown et al., 2022). This study sought to systematically review the effectiveness of interventions to mitigate professional caregivers’ stress. Ten studies met the eligibility criteria, although only seven provided sufficient data to be included in the analysis. Compared to usual care, interventions can mitigate stress and burden not attributed to client behaviour (g = −.85, *p* < .001) and can possibly improve self-efficacy (g = −.35, *p* = .07). The strongest evidence exists for organisation focused interventions (g = −.58, *p* = .08), although this should be cautiously interpreted given the small number of included studies and inability to measure the size of variance between subgroups that did not contain independent data.

### Mitigating stress and burden

Stress and burden are terms used interchangeably to describe a modifiable psychological state of feeling “stressed out” (McEwen, 2005). The subgroup analysis results of this study indicated that interventions can mitigate professional caregivers’ stress and burden that is not specifically attributed to client behaviour. Supporting this finding, an eligible study which had insufficient data to include in the meta-analysis found no significant reduction in burden related to clients’ dementia-specific challenging behaviour in either intervention group (Halek et al., 2020). Although few intervention studies measure caregivers’ self-reported psychological stress and burden that relates to client behaviour, of those identified, none report significant intervention effects (Bramble et al., 2011; Dichter et al., 2017; Wells et al., 2000). On the other hand, intervention studies that measure professional caregivers’ self-reported stress and burden which are not related to client behaviour have demonstrated significant reductions in caregivers’ stress and burden (Borbasi et al., 2011; Davison et al., 2007). This may suggest measures of stress related to client behaviour are not sensitive to detect change or that interventions that target client behaviour alone are not effective at mitigating occupational stress and burden in professional caregivers.

While agitation, apathy and depressive behaviours can be distressing for caregivers (Feast et al., 2016), positive relationships with clients (Quinn et al., 2009), the working environment (Savundranayagam et al., 2021), and organisational culture and support (Chamberlain et al., 2017) may moderate or buffer the effects of stress. In addition, one of the greatest predictors of burnout may be low self-efficacy (Duffy et al., 2009). Although not statistically confirmed, the results of this study suggest that interventions can increase caregivers’ self-efficacy, which may be a contributing factor in the mitigation of stress and burden not related to client behaviour. This may explain why non-client-specific outcome measures appear more sensitive to intervention effects and aligns with McEwen’s (2005) theory that to mitigate stress, interventions should optimise moderating factors that promote adaptation, rather than simply seeking to reduce stressors. Our results suggest interventions for professional caregiver stress should seek to address more than the job demands associated with client behaviour that caregivers find challenging.

### Mitigating burnout

While interventions may improve self-efficacy, a protective factor against burnout (Maslach & Jackson, 1981), the results of this study found no evidence that interventions can mitigate emotional exhaustion or depersonalisation which are primary burnout risk factors in professional dementia caregivers. While often conceptualised in the same framework as stress, burnout is typically associated with chronic stress (Maslach & Jackson, 1981) and locates an individual’s stress experience within the organisational context of their work (Maslach et al., 2001). Supporting our finding, one study found stress interventions less effective for employees with high levels of baseline stress compared to lower levels (Van der Klink et al., 2001). Personal factors including self-efficacy (Duffy et al., 2009) and perceived mastery and control (Testad et al., 2010); and organisational factors including culture, resources, support and environment (Chamberlain et al., 2017; Costello, Walsh, et al., 2019; Woodhead et al., 2016) are associated with burnout. Three of the five burnout interventions included in this analysis trialled caregiver training interventions which may be insufficient to mitigate burnout without continued leadership support and cultural improvement to support the implementation of new knowledge (Chamberlain et al., 2017). Consistent with this, one eligible study with insufficient data to include in the meta-analysis found no significant effect of a caregiver education-focused intervention on any burnout domains (Zwijsen et al., 2015), while another found two organisation-focused interventions significantly reduced work-related burnout (Halek et al., 2020). It has been suggested that burnout risk in professional caregivers is not as high as generally accepted (Costello, Cooper, et al., 2019; de Rooij et al., 2012) and that interventions may need to have a more individualised treatment approach (Kuske et al., 2009). While this may explain some of the results of this review, there were an insufficient number of studies to conduct additional analysis on intervention types. Further research is required to examine the effect of interventions on caregiver burnout and research may need to focus on caregivers identified at higher risk of burnout.

### Effect of intervention type

Our results are consistent with recent systematic review findings on the effectiveness of client-focused (directed towards person living with dementia) interventions on family caregivers burden and distress that found large variation in the results and recommended further research (Feast et al., 2016). Some non-randomised trials with professional caregivers have found training interventions to mitigate stress, burden and burnout (Borbasi et al., 2011; Wells et al., 2000), while others report no intervention effects (Davison et al., 2007). Further research is required to determine if, what, and potentially how client and caregiver interventions mitigate professional caregivers’ stress. Conversely, our finding that organisation-focused interventions may mitigate professional caregivers’ stress, conflicts with generalised occupational stress literature, which proposes that organisation-focused interventions are least effective for participants from a wide range of occupations (Richardson & Rothstein, 2008). Since caregivers’ stressors can relate to the functioning of the workplace, and to interactions between caregivers and immediate co-workers (Cohen-Mansfield, 1995), organisational interventions that address social climate, social resources, teamwork, and leadership styles may be effective in this population. Supporting this, interventions from two eligible studies with insufficient data for meta-analysis used a case conferencing strategy to provide social support within the organisation and found large and significant intervention effects (Halek et al., 2020). Existing evidence supports the moderating effects of social support on health-care workers’ occupational stress and burnout (Cohen-Mansfield, 1995; Woodhead et al., 2016). Our finding that caregivers may benefit from employers systematically implementing holistic interventions that improve personal and organisational resources may be optimised by improving caregivers perceived social support within the organisation.

### Limitations

Notwithstanding these findings, this review has limitations. Studies eligible to be included in this review included randomised controlled trials published in peer-reviewed journals in English language only which may lead to publication bias. Negative results are less likely to be published, thus this review may inadequately represent all research conducted in this field. However, included studies were conducted in English and non-English speaking countries, suggesting generalisability to multiple cultural contexts. This meta-analysis investigated results immediately post intervention to maintain comparable data; thus, long-term effects were not examined.

Another limitation of this review is the use of change scores and imputed missing data. While some research suggests meta-analysis of change scores may inflate the significance of the results compared to meta-analysis of follow-up scores (Fu & Holmer, 2016), meta-analysis of change scores is common practice (Higgins et al., 2022). Furthermore, imputation of change scores and standard deviations is considered better practice than omitting studies entirely (Weir et al., 2018). Nonetheless, imputing change scores meant ANCOVA-adjusted change scores accounting for baseline measures as a covariant were not available for most studies included in this review. This does add a degree of bias into these results and as such they should be cautiously interpreted.

## Conclusion

The effects of caregiver stress can negatively impact the physical and mental health of professional caregivers and people living with dementia in RACFs. There is an opportunity for residential aged care employers to reduce stress and burden and improve self-efficacy in professional caregivers, by implementing interventions that improve caregivers’ personal and organisational resources, possibly by prioritising organisational support strategies. Still, there remains a need for more well-designed intervention trials as well as synthesis studies to determine the types of interventions and change mechanisms that are effective at mitigating caregiver stress. Future research should also examine what interventions may be effective in treating burnout in high-risk professional dementia caregivers and explore longitudinal effects. Given the current workforce challenges to recruit and retain caregivers, and the economic and health costs of occupational stress, interventions implemented by RACF employers may be the key to sustaining a healthy and capable professional caregiver workforce.
